# Race and Ethnicity Impacts Overall Survival of Patients with Appendiceal Cancer Who Undergo Cytoreductive Surgery with Hyperthermic Intraperitoneal Chemotherapy

**DOI:** 10.3390/cancers15153990

**Published:** 2023-08-06

**Authors:** Devon C. Freudenberger, Vignesh Vudatha, Luke G. Wolfe, Andrea N. Riner, Kelly M. Herremans, Brian K. Sparkman, Leopoldo J. Fernandez, Jose G. Trevino

**Affiliations:** 1 Department of Surgery, Virginia Commonwealth University School of Medicine, Richmond, VA 23298, USA; devon.freudenberger@vcuhealth.org (D.C.F.); vignesh.vudatha@vcuhealth.org (V.V.); luke.wolfe@vcuhealth.org (L.G.W.); brian.sparkman@vcuhealth.org (B.K.S.); leopoldo.fernandez@vcuhealth.org (L.J.F.); 2 Department of Surgery, University of Florida College of Medicine, Gainesville, FL 32610, USA; andrea.riner@surgery.ufl.edu (A.N.R.); kelly.herremans@surgery.ufl.edu (K.M.H.)

**Keywords:** appendiceal cancer, cytoreductive surgery (CRS), hyperthermic intraperitoneal chemotherapy (HIPEC), racial disparities, cancer survivorship

## Abstract

**Simple Summary:**

The influence of race/ethnicity on overall survival in patients with appendiceal cancer treated with cytoreductive surgery and hyperthermic intraperitoneal chemotherapy is unknown. In this first large-scale study, we demonstrate that patient race/ethnicity plays a role in overall survival in this patient population. Striking differences in patient sociodemographics, including patient age, sex, income, education, and geographic location, may contribute to these disparities. However, no differences in patient perioperative and postoperative outcomes were found (e.g., tumor grade, margins, hospital length of stay, readmission rates, and 30/90-day mortality). Despite this, when compared by race/ethnicity, patients of non-Hispanic Black descent had worse overall survival rates than patients of Hispanic descent. Non-Hispanic White individuals had similar overall survival rates to non-Hispanic Black individuals. Further inquiry is warranted to determine why this survival disparity is present amongst diverse patient groups afflicted with this disease.

**Abstract:**

Appendiceal cancer treatment may include cytoreductive surgery and hyperthermic intraperitoneal chemotherapy (CRS/HIPEC). We investigated whether patient race/ethnicity influences outcomes and overall survival for patients with appendiceal cancer who undergo CRS/HIPEC. We queried the National Cancer Database for adult patients with appendiceal cancer treated with CRS/HIPEC from 2006 to 2018. Patients were stratified by race/ethnicity: non-Hispanic White (NHW), non-Hispanic Black (NHB), Hispanic, and Other. Sociodemographics and outcomes were compared using descriptive statistics. Kaplan–Meier survival analysis and Log-rank tests assessed differences in overall survival (OS). Cox Multivariate Regression evaluated factors associated with OS. In total, 2532 patients were identified: 2098 (82.9%) NHW, 186 (7.3%) NHB, 127 (5.0%) Hispanic, and 121 (4.8%) Other patients. The sociodemographics were statistically different across groups. The perioperative and postoperative outcomes were similar. OS was significantly different by race/ethnicity (*p* = 0.0029). NHB patients compared to Hispanic patients had the shortest median OS (106.7 vs. 145.9 months, *p* = 0.0093). Race/ethnicity was independently associated with OS: NHB (HR: 2.117 [1.306, 3.431], *p* = 0.0023) and NHW (HR: 1.549 [1.007, 2.383], *p* = 0.0463) patients compared to Hispanic patients had worse survival rates. Racial/ethnic disparities exist for patients with appendiceal cancer undergoing CRS/HIPEC. Despite having similar tumor and treatment characteristics, OS is associated with patient race/ethnicity.

## 1. Introduction

Racial and ethnic disparities in incidence and outcomes are well documented across the spectrum of cancer. These disparities can be the result of structural and socioeconomic factors, including the lack of clinical trial representation [1]. These inequities are present in a wide range of cancer types including complex gastrointestinal malignancies [2]. While sociodemographic factors have been analyzed in the context of advanced cancers such as pancreatic or colon cancer, there have been limited studies assessing their impact on patients with peritoneal carcinomatosis [3,4].

Peritoneal carcinomatosis, or peritoneal surface malignancy, is the metastasis of cancer to the peritoneal lining of the abdominal cavity. These cancerous implants can occur as a primary peritoneal mesothelioma or secondary to spread from primary abdominal or gynecologic malignancies. Peritoneal carcinomatosis from gastrointestinal or gynecologic primary sources represents advanced Stage IV disease and projects a poor prognosis to patient survival. Fortunately, the introduction of cytoreductive surgery (CRS) and hyperthermic intraperitoneal chemotherapy (HIPEC) as a selective treatment modality has improved patient outcomes, including 5-year survival [5,6]. The procedure carries a high morbidity rate and requires the coordination of multiple services. Yet, it has been shown to have a lower morbidity rate than other advanced oncologic procedures, such as esophagectomy and hepatectomy [7]. During this surgical procedure, macroscopic disease is resected to less than 2 mm and various anatomic resections are performed, including peritonectomy, cholecystectomy, colectomy, omentectomy, etc., specific to the patient’s disease involvement. Once cytoreduction is complete, the peritoneal cavity is infused with heated chemotherapy to address any remaining microscopic disease [8]. Given the complexity and morbidity associated with this procedure, understanding which patients may be disproportionately impacted by this disease and/or may benefit from this aggressive treatment option is key to improving care in this patient cohort.

There have been very few studies that have assessed health and social disparities, particularly race and ethnicity, in patients with peritoneal carcinomatosis treated with CRS/HIPEC. Furthermore, the studies that have investigated health disparities in this patient population are limited by their small sample sizes and comparison across heterogenous cancer types (i.e., colon, gastric, ovarian, appendiceal, etc.) [9,10,11,12,13]. In the present study, we chose appendiceal cancer as the singular disease to investigate. Appendiceal cancer with peritoneal surface involvement is the most well-documented disease process for which CRS/HIPEC has become a standard treatment option [14], allowing for a larger sample population to study. The focus of this study was to determine if racial/ethnic disparities exist for the outcomes and survival of patients with appendiceal cancer managed with CRS/HIPEC. We hypothesized that racial disparities exist in this cohort, similar to other advanced gastrointestinal cancers [2,15].

## 2. Materials and Methods

### 2.1. Patient Case Identification

For this cross-sectional observation study, data were obtained from the National Cancer Database (NCDB) 2019 Participant User File (PUF). The NCDB is a joint project of the Commission on Cancer of the American College of Surgeons and the American Cancer Society. The NCDB is a hospital-based nationwide comprehensive clinical registry that captures 72% of all newly diagnosed malignancies in the United States annually. All data used in this study were derived from a de-identified NCDB file. Methods of data collection have been described elsewhere [16,17]. This study did not require a review by the institutional review board. This study was completed in compliance with the Strengthening the Reporting of Observational Studies in Epidemiology (STROBE) reporting guidelines [18].

All adult patients, 18–89 years old, diagnosed with appendiceal cancer were identified with ICD-10 code C18.1 (malignant neoplasm of the appendix) from the years 2006 to 2018. Of note, patients diagnosed in 2019 were excluded, as survival endpoints, the primary outcome of this study, were missing in all cases. To identify which of these patients underwent CRS/HIPEC, the “Systemic/Surgery Sequence” (Rx_Summ_Systemic_Sur_Seq) was used to identify HIPEC, with code 5 or 6 indicating intraoperative systemic therapy, as has been carried out in previous studies [9,19]. To verify that these patients underwent CRS, “Surgical Procedure of Primary Site” (Rx_Summ_Surg_Prim_Site) was used where any code representing a surgical resection was used to represent CRS, and code 00 represented no surgery, which was excluded.

Patient sociodemographics, tumor characteristics, and treatment outcome variables are noted in Table 1 and Table 2. Surgical margins were reclassified as R0/R1 resection, R2 resection, and resection with residual tumor not otherwise specified (NOS) in order to pseudo-measure the completeness of cytoreduction score (CC-score), a standard measure in CRS/HIPEC. In this schema, R0/R1 was used to represent a CC-score of 0, and R2 was used to represent a CC-score of ≥1. All missing data were reported as unknown.

### 2.2. Statistical Analysis

Patient cases were stratified by race/ethnicity into the following broad racial/ethnic groups: non-Hispanic White, non-Hispanic Black, Hispanic, and Other. Differences in patient sociodemographics, tumor characteristics, surgical treatment, and postoperative outcomes were determined via an X^2^ test for categorical variables and Kruskal–Wallis test for continuous variables due to the non-parametric distribution of the data.

Survival was calculated using the reported last date of contact and vital status from the database. Five-year, ten-year, and overall survival rates were calculated individually. Kaplan–Meier survival analyses with Log Rank tests were conducted to determine differences in survival by patient race/ethnic group. The median overall survival rate was determined from the 50%-point estimate for each racial/ethnic group. If a group did not reach the estimated 50% survival point, then median overall survival rate could not be calculated in this study’s follow-up timeframe. Differences in survival between individual racial/ethnic groups were compared using pair-wise tests and reported with the Sidak correction *p*-value to correct for multiple comparisons.

A Cox Proportional Hazards Regression model was conducted to assess for significant clinical and sociodemographic factors associated with overall survival. A stepwise selection procedure was performed including all variables with a *p*-value ≤ 0.2 on the univariate analysis.

All continuous variables are reported as medians, with their interquartile range, given their non-parametric distribution. All tests were two-sided, and an alpha value of 0.05 was used for assessing statistical significance. Data analyses were performed using SAS Version 9.4 (SAS Institute, Cary, NC, USA).

## 3. Results

### 3.1. Patient Sociodemographics

In total, 2532 patients with appendiceal cancer who underwent CRS/HIPEC were identified, with a distribution as noted in Table 1. For all sociodemographic variables, there were statistically significant differences between racial/ethnic patient groups. There was a significant difference in the distribution of the afflicted sex by race/ethnicity, as non-Hispanic White and Other patients were affected at nearly equal rates according to sex, but over 60% of both the non-Hispanic Black and Hispanic patient cohorts were female (*p* = 0.006). Hispanic patients were significantly younger, with a median age of diagnosis of 50 years compared to the median age of diagnosis of 59 years old for Other patients (*p* < 0.001). Non-Hispanic White patients had the highest rate of private insurance coverage (68.4%), whereas Hispanic patients had the highest rate of Medicaid coverage (16.5%), and Other patients had the highest rate of Medicare coverage (24.8%), possibly reflective of their older age (*p* < 0.001). Most patients resided in metropolitan or urban areas, but non-Hispanic White patients traveled the furthest for medical treatment (36.6 miles, *p* < 0.001). The median household income and education level varied greatly amongst the different groups, with higher incomes and education levels in the non-Hispanic White and Other groups compared to the non-Hispanic Black and Hispanic groups. There were no differences in year of diagnosis or the burden of comorbidities between groups.

### 3.2. Cancer Characteristics and Treatment Outcomes

When comparing the tumor characteristics, surgical treatment, and postoperative outcomes, there were no statistically significant differences by race/ethnicity (Table 2). Tumor grade, regardless of race/ethnicity, was similar, with half or more of patients having well-differentiated to moderately differentiated tumors. Surgical margins were also similar, with at least 60% of each patient group achieving an R0/R1 resection. Postoperatively, all patients had similar lengths of stay, readmission rates, mortality rates, and lengths of follow-up.

### 3.3. Survival Analysis

**Figure 1:**
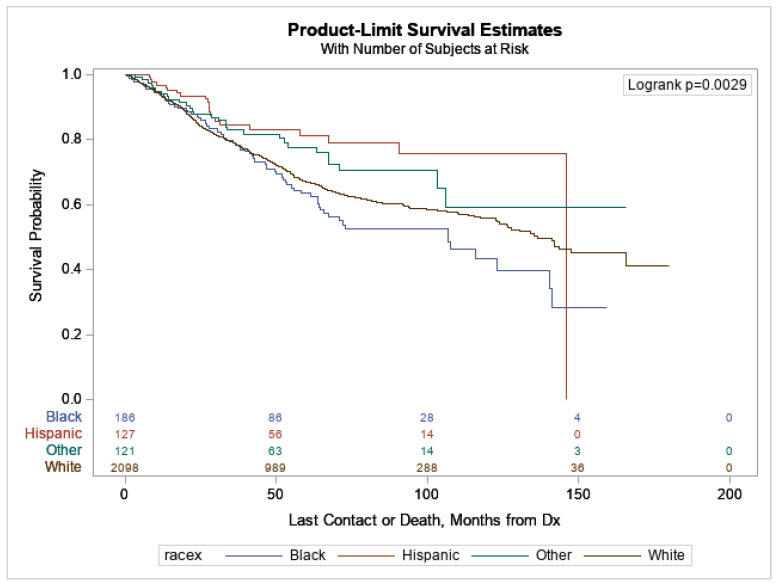
Overall survival by patient race/ethnicity. Median overall survival was reached by patients in the non-Hispanic White (136.3 months), non-Hispanic Black (106.7 months), and Hispanic (145.9 months) racial/ethnic groups, while patients in the Other racial/ethnic group did not reach median overall survival in this study’s timeframe (*p* = 0.003).

Survival analyses were completed for 5-year survival, 10-year survival, and overall survival (Figure 1). The distribution of survival was statistically different for 5-year (*p* = 0.012), 10-year (*p* = 0.004), and overall survival (*p* = 0.003) between the racial/ethnic groups. Hispanic patients had the best survival rates, and non-Hispanic Black patients consistently had the worst survival rates.

The median overall survival time was 12 years (145.9 months) for Hispanic patients, 11 years (136.3 months) for non-Hispanic White patients, and 9 (106.7 months) years for non-Hispanic Black patients. The median overall survival time was not reached by the Other group during this study’s timeframe and, therefore, could not be calculated. Specifically, when comparing groups individually, Hispanic patients had better overall survival rates compared to non-Hispanic Black patients (Sidak *p* = 0.012), but there was no difference for any other racial/ethnic groups when compared individually via multi-comparison analysis.

### 3.4. Factors Associated with Overall Survival

Cox multivariate regression was carried out to identify clinical and sociodemographic factors associated with overall survival (Table 3). Only patient age, sex, race/ethnicity, facility type, urban versus rural status, income, days to surgery, tumor grade, surgical margins, and year of diagnosis were included in the final model after meeting the inclusion criteria. Importantly, race/ethnicity was found to be significantly associated with overall survival, with non-Hispanic Black patients (HR: 2.12 [1.31, 3.43], *p* < 0.001) and non-Hispanic White patients (HR: 1.55 [1.01, 2.39] *p* = 0.05) having a statistically significantly higher risk of death when compared to Hispanic patients.

Increasing tumor grade (e.g., poorly differentiated, HR:4.52 [3.68, 5.56], *p* < 0.001) and positive surgical margins (e.g., R2 surgical resection, HR 1.96 [1.53, 2.50], *p* < 0.001) were significantly associated with worse overall survival. Female sex, living in a metropolitan area, and later year of diagnosis were found to be protective factors associated with overall survival.

## 4. Discussion

Understanding health disparities in the treatment of cancer is crucial towards providing high-quality, individual patient care. Though significant strides have been made to identify these differences across multiple cancers, there is limited literature focusing on the disparities present in the peritoneal carcinomatosis population, specifically in patients with appendiceal cancer with peritoneal surface involvement. In this study, we present an analysis of appendiceal peritoneal carcinomatosis patients with similar perioperative results by patient race/ethnicity. Despite inequities in education and income, and other important sociodemographic factors, between the different racial/ethnic groups, there were no statistically significant differences in tumor characteristics and 30-day postoperative outcomes.

However, race/ethnicity did have an impact on long-term mortality and overall survival, with non-Hispanic Black patients having the shortest median overall survival time and Hispanic patients having the longest median overall survival time. Although, it should be noted that Hispanic patients were diagnosed at a younger age than non-Hispanic Black patients, age was controlled for during the Cox multivariate regression analysis. Our results are corroborated by those reported by Holowatyj et al. who, when comparing overall survival in patients with early-onset appendiceal cancer (patients diagnosed between the ages 20 and 49) using the National Institutes of Health/National Cancer Institute’s Surveillance, Epidemiology and End Results (SEER) program, found that non-Hispanic Black individuals had a higher risk of death (HR: 1.47 [1.10–1.95], *p* = 0.009) when compared to non-Hispanic White individuals [20]. Similarly, in a recent study, it was shown that in 2019, Black individuals had the highest cancer mortality rates for all racial/ethnic groups in the United States, despite decreases in mortality over the preceding 20 years. The authors suggested that this disparity was a result of longstanding societal racial/ethnic inequities rather than differences in individual genetics and biology [21]. This reasoning, however, may not adequately explain the disparities found in appendiceal cancer, as multiple studies have shown differences exist in appendiceal tumor histology with respect to patient race/ethnicity [22,23]. It is likely that there is a complex interplay of sociodemographic, biologic, and genomic tumor-related factors that are influencing survival in this patient population [24].

As prior research has demonstrated, our results also support the phenomenon of the “Hispanic Paradox” [25,26,27,28]. The Hispanic Paradox is an epidemiologic phenomenon that Hispanic individuals in the United States have better health outcomes in certain instances compared to non-Hispanic White individuals in the United States, despite Hispanic individuals on average experiencing more socioeconomic disadvantages than their non-Hispanic White counterparts. With this demonstrated survival advantage, the markedly low Hispanic patient sample size compared to the non-Hispanic White patient sample size (Hispanic: *n* = 127 vs. Non-Hispanic White: *n* = 2098) begs the question why more Hispanic individuals are not accessing this life-extending treatment. Byrne et al. previously showed using NCDB data that non-Hispanic individuals were more likely to receive intraperitoneal chemotherapy for appendiceal cancer than Hispanic individuals (HR: 1.92 [1.21, 3.05], *p* = 0.0055) [19]. Given that the NCDB accounts for 72% of newly diagnosed cancers, but only 1500 or so Commission-on-Cancer-accredited hospitals in the United States, it is hard to deduce whether this sample size is due to a failure to capture patients that receive treatment at non-NCDB affiliated facilities or if other disparities are contributing to the lack of access. The distance traveled to the treating facility was statistically different between the groups, with non-Hispanic White patients traveling the furthest median distance. Perhaps this variable alone reflects socioeconomic differences and disparities that contribute to access to care. For example, in a recent Medicare-based cohort study of individuals in the United States 65 years and older with colorectal peritoneal metastases, the authors demonstrated that living further away from an HIPEC center and higher measured social vulnerability were independently associated with lower odds of accessing CRS/HIPEC [29]. This, along with our data, advocates for increased CRS/HIPEC access and the treatment of appendiceal cancer for diverse patient populations.

The limitations of this study include the limitations inherent to studies utilizing large databases. Missing data was a particular challenge in this study, with some variables, such as the sociodemographic variables of income and education level, missing up to 16% of data. To handle this issue, all missing data were recoded as unknown to prevent the loss of patient cases given the smaller sample size of this patient population. Despite using the most up-to-date 2019 PUF, all 2019 data were excluded from this study as survival data were missing.

Importantly, specific to the procedure of CRS/HIPEC, NCDB does not have a variable for PCI (peritoneal cancer index), which is an important prognostic factor in this disease process and treatment [30,31,32]. Additionally, there is no measure for completeness of cytoreduction (e.g., CC-score), a measure of the reduction in visible disease during the cytoreductive portion of the procedure that is intraoperatively determined by the operating surgeon. Again, this is another important prognostic factor [33,34]. The CC-score was extrapolated with the provided surgical margins (R0, R1, etc.), where R0/R1 represented a CC-score of 0 (i.e., no grossly visible disease present) and R2 pseudo-represented a CC-score of ≥1 (i.e., grossly visible tumor present), but this is an assumption of the true intraoperative CC score.

Perioperative complications, beyond 30- and 90-day mortality and 30-day readmission, are not recorded by the NCDB, so we were unable to assess differences in the incidence of complications by race/ethnicity, something that has been shown to be significant in other gastrointestinal malignancies [1,2,4,35,36,37,38]. Lastly, there was a substantial difference in the sample sizes of the different racial/ethnic groups, which means they were not representative of the racial/ethnic breakdown of the United States according to United States Census estimates, which could have resulted in heavily biased results [39]. Also, given the small sample sizes, a more granular breakdown of individual race/ethnicity (e.g., Cuban vs. Dominican) was not possible, requiring the creation of broader racial/ethnic groups that may misrepresent the more specific impact of race/ethnicity on survival. However, despite these limitations, this is the largest study to date examining racial/ethnic disparities in survival in this patient population.

## 5. Conclusions

Patient race/ethnicity is a statistically significant factor associated with overall survival for patients with appendiceal cancer and peritoneal surface disease who undergo CRS/HIPEC. Hispanic individuals experience the best overall survival rate, whereas non-Hispanic Black individuals experience the worst overall survival rate. Sociodemographic differences are strikingly apparent in this patient population. However, it is uncertain at this time exactly what factors and to what extent these factors are contributing to these apparent disparities in survivorship after CRS/HIPEC. Social determinants of health and differences in tumor biology and genomics may be drivers of differences in survival. Further inquiry into these is warranted to address the differences in survival for diverse patient populations with appendiceal cancer undergoing CRS/HIPEC in order to mitigate negative drivers of survival.

## Figures and Tables

**Table 1:** Patient sociodemographic characteristics by race/ethnicity (*n* = 2532).

Variable	Non-Hispanic White (*n* = 2098)	Non-Hispanic Black (*n* = 186)	Hispanic (*n* = 127)	Other (*n* = 121)	*p*-Value
**Sex** Male Female	999 (47.6%) 1099 (52.4%)	71 (38.2%) 115 (61.8%)	45 (35.4%) 82 (64.6%)	57 (47.1%) 64 (52.9%)	0.006
**Age (y)**	57.0 [48.0, 65.0]	55.0 [44.0, 62.0]	50.0 [40.0, 61.0]	59.0 [49.0, 65.0]	<0.001
**Insurance** Private Medicaid Medicare Other Govt Uninsured Unknown	1435 (68.4%) 69 (3.3%) 497 (23.7%) 40 (1.9%) 28 (1.3%) 29 (1.4%)	109 (58.6%) 19 (10.2%) 35 (18.8%) 14 (7.5%) 5 (2.7%) 4 (2.2%)	71 (55.9%) 21 (16.5%) 24 (18.9%) 3 (2.4%) 6 (4.7%) 2 (1.6%)	71 (58.7%) 11 (9.1%) 30 (24.8%) 3 (2.5%) 4 (3.3%) 2 (1.7%)	<0.001
**Distance (miles)**	36.6 [11.7, 122.3]	25.0 [6.4, 96.2]	19.8 [7.3, 91.0]	17.9 [7.7, 47.0]	<0.001
**Urban vs. Rural** Metro Urban Rural Unknown	1519 (72.4%) 220 (10.5%) 31 (1.5%) 328 (15.6%)	146 (78.5%) 9 (4.8%) 2 (1.1%) 29 (15.6%)	113 (89.0%) 6 (4.7%) 0 (0.0%) 8 (6.3%)	105 (86.8%) 3 (2.5%) 0 (0.0%) 13 (10.7%)	<0.001
Facility Type * CCP CCCP ARP INCP Unknown	28 (1.3%) 367 (17.5%) 1260 (60.1%) 217 (10.3%) 226 (10.8%)	3 (1.6%) 26 (14.0%) 106 (57.0%) 19 (10.2%) 32 (17.2%)	1 (0.8%) 11 (8.7%) 67 (52.8%) 23 (18.1%) 25 (19.7%)	2 (1.7%) 26 (21.5%) 73 (60.3%) 13 (10.7%) 7 (5.8%)	0.001
**Location** Atlantic Central Mountain New England Pacific Unknown	848 (40.4%) 632 (30.1%) 69 (3.3%) 120 (5.7%) 203 (9.7%) 226 (10.8%)	99 (53.2%) 37 (19.9%) 1 (0.5%) 6(3.2%) 11 (5.9%) 32 (17.2%)	42 (33.1%) 24 (18.9%) 4 (3.2%) 5 (3.9%) 27 (21.3%) 25 (19.7%)	44 (36.4%) 33 (27.3%) 2 (1.7%) 4 (3.3%) 31 (25.6%) 7 (5.8%)	<0.001
**% without High School** **Degree** ≥17.6% 10.9–17.5% 6.3–10.8% <6.3% Unknown	219 (10.4%) 382 (18.2%) 553 (26.4%) 601 (28.7%) 343 (16.4%)	49 (26.3%) 50 (26.9%) 32 (17.2%) 21 (11.3%) 34 (18.3%)	47 (37.0%) 19 (15.0%) 26 (20.5%) 14 (11.0%) 21 (16.5%)	19 (15.7%) 21 (17.4%) 29 (24.0%) 34 (28.1%) 18 (14.9%)	<0.001
**Median Income** <USD 40,227 USD 40,227–50,353 USD 50,354–63,332 >USD 63,333 Unknown	203 (9.7%) 305 (14.5%) 453 (21.6%) 789 (37.6%) 348 (16.6%)	52 (28.0%) 38 (20.4%) 29 (15.6%) 32 (17.2%) 35 (18.8%)	19 (15.0%) 28 (22.0%) 24 (18.9%) 35 (27.6%) 21 (16.5%)	8 (6.6%) 7 (5.8%) 29 (24.0%) 59 (48.8%) 18 (14.9%)	<0.001
**Year of Diagnosis** 2006–2010 2011–2014 2015–2018	535 (25.5%) 639 (30.5%) 924 (44.0%)	44 (23.7%) 51 (27.4%) 91 (48.9%)	22 (17.3%) 38 (29.9%) 67 (52.8%)	28 (23.1%) 41 (33.9%) 52 (43.0%)	0.29
**Charlson–Deyo** 0 1 2 ≥3	1779 (84.8%) 256 (12.2%) 40 (1.91%) 23 (1.1%)	149 (80.1%) 26 (14.0%) 8 (4.3%) 3 (1.6%)	107 (84.3%) 13 (10.2%) 5 (3.9%) 2 (1.6%)	94 (77.7%) 20 (16.5%) 6 (5.0%) 1 (0.8%)	0.12

* CCP: Community Cancer Program, CCCP: Comprehensive Community Cancer Program, ARP: Academic/Research Program, INCP: Integrated Network Cancer Program.

**Table 2:** Patient tumor characteristics and treatment outcomes by race/ethnicity.

Variable	Non-Hispanic White (*n* = 2098)	Non-Hispanic Black (*n* = 186)	Hispanic (*n* = 127)	Other (*n* = 121)	*p*-Value
**Tumor Grade** Well-differentiated Moderately differentiated Poorly differentiated Undifferentiated Undetermined Unknown	692 (33.0%) 445 (21.2%) 277 (13.2%) 49 (2.3%) 395 (18.8%) 240 (11.4%)	61 (32.8%) 40 (21.5%) 18 (9.7%) 2 (1.0%) 39 (21.0%) 26 (14.0%)	45 (35.4%) 37 (29.1%) 10 (7.9%) 0 (0.0%) 20 (15.8%) 15 (11.8%)	38 (31.4%) 30 (24.8%) 11 (9.1%) 3 (2.5%) 24 (19.8%) 15 (12.4%)	0.39
**Margins** R0/R1 R2 Residual, NOS * Unknown	1276 (60.8%) 129 (6.2%) 229 (10.9%) 464 (22.1%)	121 (65.1%) 17 (9.1%) 19 (10.2%) 29 (15.6%)	77 (60.6%) 7 (5.5%) 5 (3.9%) 38 (29.9%)	72 (59.5%) 9 (7.4%) 12 (9.9%) 28 (23.1%)	0.065
**Hospital LOS (d)**	9.0 [6.0, 14.0]	9.0 [7.0, 13.0]	10.0 [6.0, 15.0]	9.0 [6.0, 15.0]	0.96
**Readmission** None Unplanned Planned Both Unknown	1879 (89.6%) 130 (6.2%) 21 (1.0%) 3 (0.1%) 65 (3.1%)	162 (87.1%) 14 (7.5%) 3 (1.6%) 0 (0.0%) 7 (3.8%)	112 (88.2%) 9 (7.1%) 4 (3.2%) 0 (0.0%) 2 (1.6%)	110 (90.9%) 6 (5.0%) 3 (2.5%) 0 (0.0%) 2 (1.6%)	0.59
**30 d Mortality**	18 (0.9%)	4 (2.2%)	0 (0.0%)	0 (0.0%)	0.36
**90 d Mortality**	57 (2.8%)	5 (2.7%)	0 (0.0%)	2 (1.7%)	0.64
**Length of Follow-Up (m)**	47.0 [27.2, 76.7]	46.2 [26.7, 71.8]	45.1 [27.4, 72.4]	51.1 [30.0, 79.2]	0.80
**Overall Mortality**	688 (32.8%)	73 (39.3%)	22 (17.3%)	29 (24.0%)	<0.001

* NOS: not otherwise specified.

**Table 3:** Cox multivariate regression for factors associated with overall survival. Stepwise multivariate analysis with inclusion of variables if *p* < 0.2 on univariate analysis. Variables of patient sex, race, age, days to surgery, facility type, income, urban versus rural status, tumor grade, surgical margins, and year of diagnosis met inclusion.

Variable	Hazard Ratio	95% Confidence Interval		*p*-Value
**Age**	1.02	1.02	1.03	<0.001
**Female**	0.84	0.73	0.97	0.015
**Race**				
Non-Hispanic Black	2.12	1.31	3.43	0.002
Non-Hispanic White	1.55	1.01	2.38	0.046
Other	1.09	0.62	1.92	0.76
**Facility Type**				
CCP	0.48	0.23	1.01	0.054
CCCP	1.19	0.99	1.43	0.07
INCP	1.13	0.88	1.45	0.33
**Urban vs. Rural**				
Metro	0.79	0.63	1	0.05
Rural	0.57	0.26	1.24	0.16
**Median Income**				
<USD 40,227	1.01	0.78	1.29	0.96
USD 40,227–50,353	1.13	0.91	1.41	0.26
USD 50,354–63,332	1.46	1.21	1.76	<0.001
**Days to Surgery from Diagnosis**	1	1	1.01	<0.001
**Tumor Grade**				
Moderately differentiated	1.77	1.43	2.19	<0.001
Poorly differentiated	4.52	3.68	5.56	<0.001
Undifferentiated	3.16	2.07	4.82	<0.001
Undetermined	1.96	1.59	2.41	<0.001
**Margins**				
R2	1.96	1.53	2.5	<0.001
Residual, NOS *	2.04	1.67	2.49	<0.001
Unknown	1.4	1.17	1.68	0.021
**Year of Diagnosis**				
2011–2014	0.82	0.69	0.97	0.021
2015–2018	0.67	0.55	0.82	0.001

* NOS: not otherwise, specified.

## Data Availability

Publicly available datasets were analyzed in this study. These data can be found here: www.facs.org/quality-programs/cancer-programs/national-cancer-database/puf/ (accessed on 1 July 2022).
